# Duplex-Hierarchy Representation Learning for Remote Sensing Image Classification

**DOI:** 10.3390/s24041130

**Published:** 2024-02-09

**Authors:** Xiaobin Yuan, Jingping Zhu, Hao Lei, Shengjun Peng, Weidong Wang, Xiaobin Li

**Affiliations:** 1 The School of Electronic and Information Engineering, Xi’an Jiaotong University, Xi’an 710049, China; 2 The Xi’an Institute of Optics and Precision Mechanics, Chinese Academy of Sciences, Xi’an 710119, China; 3 National Key Laboratory of Human-Machine Hybrid Augmented Intelligence, Xi’an Jiaotong University, Xi’an 710049, China; 4 Institute of Artificial Intelligence and Robotics, Xi’an Jiaotong University, Xi’an 710049, China; 5 The State Key Laboratory of Astronautic Dynamics, China Xi’an Satellite Control Center, Xi’an 710043, China; 6 PLA 63768, Xi’an 710600, China; 7 The Beijing Institute of Remote Sensing Information, Beijing 100192, China

**Keywords:** remote sensing image classification, duplex hierarchy, discriminative representation, confusion score

## Abstract

Remote sensing image classification (RSIC) is designed to assign specific semantic labels to aerial images, which is significant and fundamental in many applications. In recent years, substantial work has been conducted on RSIC with the help of deep learning models. Even though these models have greatly enhanced the performance of RSIC, the issues of diversity in the same class and similarity between different classes in remote sensing images remain huge challenges for RSIC. To solve these problems, a duplex-hierarchy representation learning (DHRL) method is proposed. The proposed DHRL method aims to explore duplex-hierarchy spaces, including a common space and a label space, to learn discriminative representations for RSIC. The proposed DHRL method consists of three main steps: First, paired images are fed to a pretrained ResNet network for extracting the corresponding features. Second, the extracted features are further explored and mapped into a common space for reducing the intra-class scatter and enlarging the inter-class separation. Third, the obtained representations are used to predict the categories of the input images, and the discrimination loss in the label space is minimized to further promote the learning of discriminative representations. Meanwhile, a confusion score is computed and added to the classification loss for guiding the discriminative representation learning via backpropagation. The comprehensive experimental results show that the proposed method is superior to the existing state-of-the-art methods on two challenging remote sensing image scene datasets, demonstrating that the proposed method is significantly effective.

## 1. Introduction

Remote sensing image classification (RSIC) allocates precise semantic descriptors to aerial images. This task holds significant importance in practical applications such as natural disaster detection [1], environmental monitoring [2], and urban planning [3]. However, RSIC still faces a great challenge: large dissimilarities in the same class and small dissimilarities between different classes. Remote sensing images may contain complex structures of abundant ground objects, representing a challenge characterized by substantial intra-class dissimilarities and limited inter-class disparities. Specifically, images of the same scene may appear to be different from each other due to the complex structures of the ground objects. In like manner, images of different scenes may appear to be similar, as they may contain common ground objects or share similar semantic information. Therefore, a discriminative feature is vital to RSIC.

Early RSIC methods [4,5,6,7,8,9,10,11,12,13,14] exploited handcrafted features to describe remote sensing images, such as color histograms (CHs) [4], scale-invariant feature transformation (SIFT) [9], and gray-level co-occurrence matrices (GLCMs) [7]. However, the handcrafted feature methods cannot meet the practical application requirements due to their inadequate extraction of high-level semantic information. Furthermore, these methods are limited by the amount of time and effort that they consume.

With the growth of the current deep learning domain, CNNs have achieved superior success in the field of remote sensing image classification. Compared with traditional methods, CNNs are able to extract representative features and show promising performance. Penatti et al. [15] introduced CNNs to remote sensing image classification. Maggiori et al. [16] devised an end-to-end framework for satellite imagery classification with CNNs. Liu et al. [17] proposed a multiscale CNN method to solve the scale variation of the objects in remote sensing images. In order to allow images to be input at arbitrary sizes, Xie et al. [18] designed a scale-free CNN (SF-CNN). Castelluccio et al. [19] used pretrained networks to carry out a remote sensing scene classification task and proved that CNNs always provide excellent performance.

**Figure 1:**
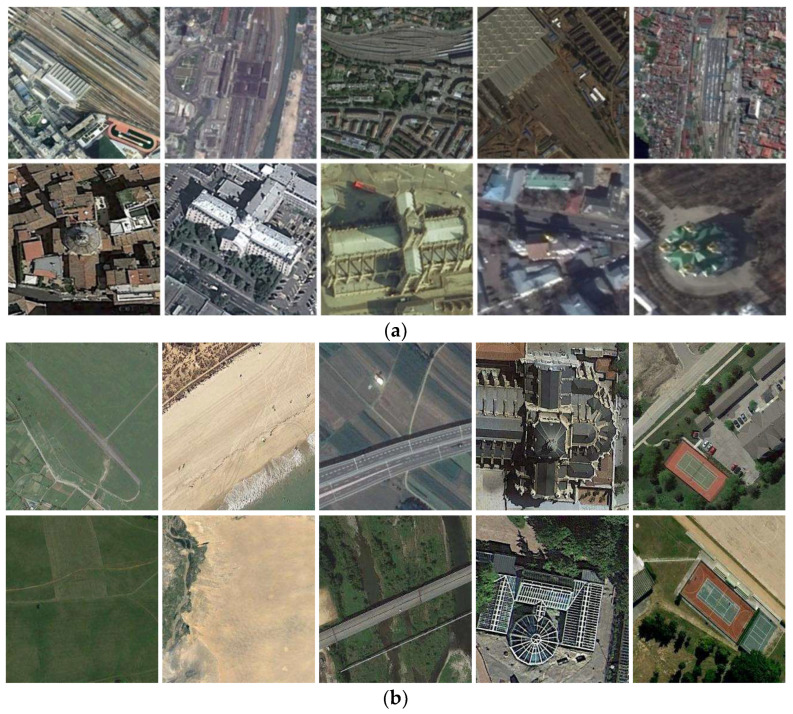
Two major challenges degenerate the scene classification performance: (**a**) intra-class diversity: railway station (the 1st row) and church (the 2nd row) [20]; (**b**) inter-class similarity: airport vs. meadow, beach vs. desert, freeway vs. bridge, palace vs. church, and tennis court vs. basketball court (from top to bottom and from left to right) [20]. This motivates us to learn more discriminative representations so that the within-class scatter is small and the between-class separation is large.

However, these CNN-based RSIC methods face an unsatisfactory classification problem: large dissimilarities in the same class and small dissimilarities between different classes. Regarding dissimilarity in the same class, the primary hurdle stems from the large variation in features appearing in the same semantic class. Images commonly differ in terms of style, shape, size, and distribution, rendering accurate scene image classification a demanding task. Several scene images from the NWPU-RESISC45 dataset [20] are shown in Figure 1. In Figure 1a, the railway stations have different shapes, and the churches present different architectural styles. The challenge of inter-class similarity is mainly due to the existence of the same objects between different scene classes or high semantic overlapping in scene classes. For instance, as shown in Figure 1b, the scene classes of both the airport and the meadow contain the same object, namely, grass, and the tennis court and basketball court contain similar semantic information.

Based on the above challenges, a duplex-hierarchy method is proposed for RSIC in this study. This method preserves the discrimination among the samples from different semantic categories for pairs of images and further improves the classification accuracy. In pursuit of this goal, this method minimizes the discrimination loss for the samples within both the label space and the common representation space. This strategy guides the model in acquiring discriminative features. Furthermore, it simultaneously minimizes the confusion loss to guide the discriminative representation learning via backpropagation. Following the method, the label information and the classification details of image pairs are both extensively utilized to guarantee that the learned representation is highly discriminative in its semantic structure. The proposed method consists of three main steps. First, paired images are fed to a pretrained ResNet [21] network for extracting the corresponding features. Second, the extracted features are further explored and mapped into a common space. Third, the obtained representation is used to predict the class label of the input image, and the loss in the label space is minimized to further facilitate the learning of the discriminative representation. At the same time, confusion scores are calculated and added to the devised confusion loss to further improve the classification accuracy.

The key contributions of this study are outlined below:

(1) An end-to-end framework is proposed for the classification of remote sensing images, where the discriminative features are learned by measuring the differences between categories in the common space and label space simultaneously.
(2) Confusion scores between categories are calculated and embedded into the designed loss function, the confusion loss, to resolve the issues of large dissimilarities in the same class and small dissimilarities between different classes in remote sensing images by minimizing the confusion loss.
(3) A large number of experiments show that the proposed method is superior to the existing state-of-the-art methods, which proves the effectiveness of the proposed method.

The remainder of this paper is organized as follows: Section 2 reviews the relevant literature. Section 3 describes the proposed method in detail. Section 4 presents the experiments. Finally, Section 5 presents the conclusions.

## 2. Related Work

The deep network approach has gained popularity in recent years, and thus far, deep learning models have achieved excellent results in numerous computer vision applications, such as image classification [22], object recognition [23], and semantic segmentation [24], and the representation of features in images has entered a new era. In contrast to handcrafted features, deep learning models have the capacity to acquire more robust, abstract representations and to differentiate features through deep architectural neural networks without requiring significant engineering skills and experiential knowledge. Among these models, convolutional neural networks (CNNs) are more applicable to classifying remotely sensed image scenes and have yielded recent results [24,25,26,27,28,29,30,31]. Generally speaking, CNN-based remote sensing image scene classification can be broadly divided into three types: pretraining-based methods, fine-tuning-based methods, and retraining-based methods.

*Pretraining-based methods.* Pretraining-based methods use pretrained networks directly for the extraction of final features from remote sensing images. In 2015, Penatti et al. [15] proposed a method for remote sensing image classification based on CNNs, and the performance of the CNNs was better than that of low-level descriptors. Hu et al. [25] extracted feature descriptors using CNNs, and the pretrained neural network models were used for scene classification. Instead of handcrafted local features, Cheng et al. [26] used off-the-shelf CNN features to construct a convolutional feature package for remote sensing classification. To leverage semantic label information, Lu et al. [27] proposed a method of aggregating features using a CNN. Methods that employ pretrained CNNs as feature extractors are relatively straightforward, feasible, and effective on small datasets.

*Fine-tuning-based methods.* These methods use fine-tuned CNNs to better extract final features from scene images, and the fully trained results are better. Liu et al. [28] coupled CNNs with a hierarchical Wasserstein loss function (HW-CNNs) to improve the discrimination ability of the CNNs. Wang et al. [29] designed an ARCNet (attentional recursive convolutional network) that could highlight critical regions and ignore non-critical regions by introducing an attentional mechanism in CNNs. Although fine-tuned CNNs can obtain better results, they are still unsuitable for the target dataset.

*Retraining-based methods.* Due to the complex spatial structures in remote sensing images, CNN models that are pretrained or fine-tuned do not effectively reflect their unique property information. Therefore, researchers have started to train neural networks from scratch on raw remote sensing image datasets. Zhang et al. [30] proposed a gradient-boosted random convolutional network (GBRCN) framework for fusing neural networks, which introduced a deep integration framework for image scene classification. He et al. [31] presented an innovative hop-connected covariance (SCCov) network to solve the problem of remote sensing classification, which could achieve superior classification performance. This method required a mass of annotated samples, but the existing remote sensing image datasets were not large enough, which could cause overfitting problems.

In this study, we used the pretrained ResNet50 as the feature extractor and directly used the feature vector from the last fully connected layer of the network as the final representation of the image.

## 3. Methods

In this section, the framework of the proposed method is introduced, followed by a detailed description of the common space and label space in representation learning and, finally, the three loss functions used in this study, namely, discrimination loss in the common space, discrimination loss in the label space, and confusion loss.

(1) Framework of DHRL

**Figure 2:**
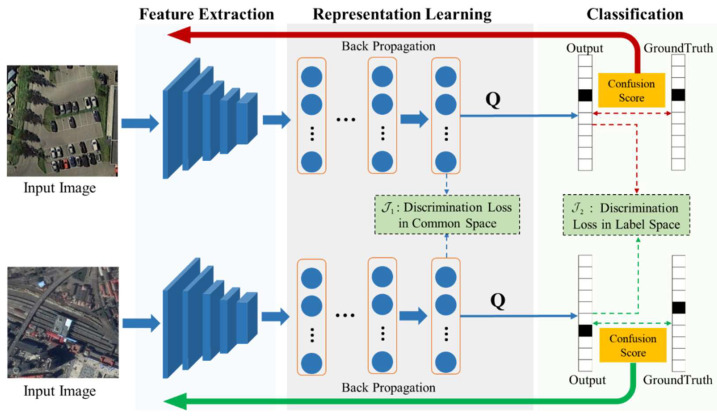
The framework of DHRL. First, the paired images are fed to a pretrained ResNet network for extracting the corresponding features. Second, the extracted features are further explored and mapped into a common representation space for reducing the intra-class scatter and enlarging the inter-class separation. Third, the obtained representation is used to predict the categories of the images and further facilitate the learning of discriminatory representations in the label space. Meanwhile, the confusion score is computed and added to the classification loss for guiding the discriminative representation learning via backpropagation.

As shown in Figure 2, the proposed framework adopts a duplex network to explore both the common space and label space to learn discriminative representations for RSIC. This method inputs a pair of images for training but only uses a single image for testing. The method consists of three main steps: First, in the feature extraction step, the semantic features of the images are extracted by the pretrained ResNet [20]. Then, the extracted features are input into the representation learning phase through several fully connected network layers to explore the consistency of the representations in the common space. Finally, relying on the assumption that common representations in the common space are optimal for classification, a linear classifier with a parameter matrix Q is used to predict the semantic categories of the input images in the label space, and the confusion score of the predicted results and the real labels is calculated using the confusion loss, which further optimizes the model.

Assume that there are n instances of image pairs, denoted as $\Psi ={\left\{\left(x_{i}^{\alpha },x_{i}^{\beta }\right)\right\}}_{i=1}^{n}$, where $x_{i}^{\alpha }$ is the input image sample and $x_{i}^{\beta }$ is another image sample of the ith instance. $x_{i}^{\alpha }$ and $x_{i}^{\beta }$ are two randomly selected images from the dataset. If the image pairs have consistent labels, the features are constrained to be as similar as possible, but for image pairs with inconsistent labels, the features are constrained to be as different as possible. This enables the same category clusters to be more compact while allowing different category clusters to be as dispersed as possible. Each pair of instances $\left(x_{i}^{\alpha },x_{i}^{\beta }\right)$ is assigned a semantic label vector $\left(y_{i}^{\alpha },y_{i}^{\beta }\right)$, and $y_{i}=\left[y_{1i},y_{2i},\ldots ,y_{ci}\right]\in R^{c}$, where *c* is the number of categories. If the *i*th instance belongs to the *j*th category, $y_{ij}=1$; otherwise, $y_{ij}=0$. Representation learning involves learning two functions for two inputs:

(1) $$u_{i}=f\left(x_{i}^{\alpha },{ϒ}_{a}\right)\in R^{d},v_{i}=f(x_{i}^{\beta },{ϒ}_{\beta })\in R^{d}$$

where d is the dimensionality of the representation in the common representation space, $u_{i}$ and $v_{i}$ are the representations of instances $x_{i}^{\alpha }$ and $x_{i}^{\beta }$ in the common space, and ${ϒ}_{a}$ and ${ϒ}_{\beta }$ are the trainable parameters of the two functions. This means that the similarity of samples from the same category is larger than the similarity of samples from different categories in the common space. In the following, the image representation matrix and the label matrix for all instances of Ψ are denoted as $U=[u_{1},u_{2},\ldots ,u_{n}]$, $V=[v_{1},v_{2},\ldots ,v_{n}]$, $Y^{\alpha }=[y_{1}^{\alpha },y_{2}^{\alpha },\ldots ,y_{3}^{\alpha }]$, and $Y^{\beta }=[y_{1}^{\beta },y_{2}^{\beta },\ldots ,y_{3}^{\beta }]$.

(2) Implementation Details

Deep neural networks are used to extract features directly from raw images, and various recent studies have shown that several excellent neural networks can be useful in RSIC tasks, such as AlexNet [32], VGG [33], and ResNet [20]. ResNet was proposed by He et al. in 2015 as a residual network. Usually, the deeper the network, the greater the amount of information that can be obtained and the richer the features. However, as the network gets deeper, it can cause problems such as gradient disappearance and gradient explosion. ResNet is an ultra-deep neural network, as it learns the residual representations between inputs and outputs, unlike the usual CNNs (AlexNet, VGG, etc.) that use participant layers to try to directly learn the mapping between inputs and outputs, and it greatly improves the accuracy; therefore, we chose ResNet as the backbone network for our proposed method.

ResNet consists of a diverse range of network layer depths. The most commonly encountered ones are 50 layers, 101 layers, and 152 layers; they are all constructed by stacking the previously mentioned residual modules together. In this study, the pretrained ResNet was used to extract the features of the conv5 layer from the original image, and features of 1×1×1204 were finally obtained after pooling.

Due to the existence of intra-class diversity and inter-class similarity in RSIC, the main solution is to make instances of different classes as separate as possible while making instances of the same class as close as possible; therefore, it is necessary to measure the content similarity between different samples. Representation learning is an attempt to find a function that maps the obtained data samples into a common space where the similarity between them can be directly measured.

In the common space, samples from the same category should be similar, while samples from different categories should be dissimilar, as the similarity of samples from the same category is greater than the similarity of samples from different categories. Therefore, common representations need to be obtained, which are learned through several fully connected network layers after obtaining the features of images. By minimizing the discrimination loss in the common space, the intra-class distance is reduced while the inter-class distance is increased. The discrimination loss of all samples from both images in the common space is measured directly:

(2) $$J_{1}=\frac{1}{n^{2}}\sum_{i,j=1}^{n}\left(\log\left(1+e^{\Lambda_{ij}}\right)-P_{\alpha \beta }\Lambda_{ij}\right)\;+\frac{1}{n^{2}}\sum_{i,j=1}^{n}\left(\log\left(1+e^{{Τ}_{ij}}\right)-P_{\alpha \alpha }{Τ}_{ij}\right)\;\;+\frac{1}{n^{2}}\sum_{i,j=1}^{n}\left(\log\left(1+e^{\Upsilon_{ij}}\right)-P_{\beta \beta }\Phi_{ij}\right)$$

where $\Lambda_{ij}=\frac{1}{2}\cos\left(u_{i},v_{j}\right)$, ${Τ}_{ij}=\frac{1}{2}\cos\left(u_{i},\mu_{j}\right)$, $\Phi_{ij}=\frac{1}{2}\cos\left(\nu_{i},v_{j}\right)$, *cos*(∙) is a cosine function used to compute the similarity of two input vectors, $P_{\alpha \beta }=L\left\{u_{i},v_{j}\right\}$, $P_{\alpha \alpha }=L\left\{u_{i},\mu_{j}\right\},\;P_{\beta \beta }=L\left\{\nu_{i},v_{j}\right\}$, and L(⋅) is 1 when the two samples are representations of intra-class samples and 0 otherwise. Each term of Equation (2) is the negative log-likelihood of the sample similarities, and minimizing the negative log-likelihood is equivalent to maximizing the likelihood. It can be seen that the larger the similarities cos(⋅) are, the larger *p*(1|*u*, *v*) will be, which means that the image samples should be classified as similar, and vice versa. The first term of Equation (2) measures the similarities between the two image samples, and the second and third terms measure the similarities between samples of the interior of the image. Therefore, Equation (2) is a reasonable measure of similarity for common representations and is an effective criterion for learning discriminative features.

**Figure 3:**
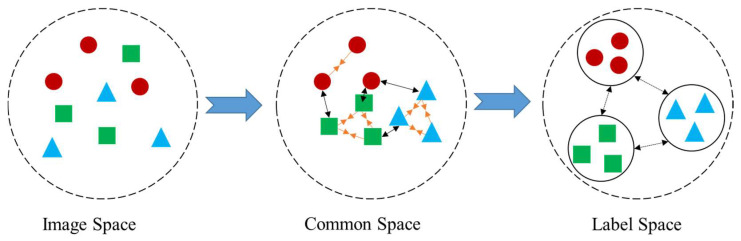
Samples in dual-level spaces. In the common space, samples with similar features are brought together and samples with different features are separated, thereby increasing the inter-category distance and decreasing the intra-category distance.

The label space is mainly used to classify the obtained representations. Due to the supervised methods, label information is used to distinguish samples from different semantic categories in order to learn more differentiated generic representations. Figure 3 demonstrates a simple example for the representations in three spaces. To preserve the discrimination of samples from different categories after the feature projection, it is assumed that the common representations are ideal for classification, and a linear classifier with a parameter matrix Q is used to predict the labels of the samples projected in the common space. This classifier takes the representations of the training data in the common space and generates a predicted label of a c-dimensional vector for each sample. For the image sample $x_{i}$, the input of linear classifier Q is the learned feature representation $u_{i}$ in the common space, and the output of linear classifier Q is the predicted label $y_{i}$. In this study, the following objective function was used to measure the discriminative loss in the label space:

(3) $$J_{2}=\frac{1}{n}||Q^{T}U-Y^{\alpha }|{|}_{F}+\frac{1}{n}||Q^{T}V-Y^{\beta }|{|}_{F}$$

where $||\cdot |{|}_{F}$ is the Frobenius norm, and Q denotes the projection matrix of the linear classifier.

To better allow the ground truth to be supervised, confusion loss is proposed in this study. The purpose of the confusion loss is to ensure that the predicted labels are closer to the true labels, especially for samples that can easily be predicted incorrectly. By calculating the confusion between them via backpropagation to the loss function, the loss can adjust the model so that it gives more attention to the samples that can easily be predicted incorrectly, minimizing the confusion loss and learning more discriminative features via backpropagation to achieve more discriminative representations. The following gives the confusion loss:

(4) $$L_{c}=(1-\sum_{k=1}^{n}\frac{e^{{y_{k}}^{T}y_{k}^{*}}}{\sum_{l=1}^{n}e^{{y_{l}}^{T}y_{l}^{*}}})\times (-\sum_{k=1}^{n}{y_{k}}^{T}log(\frac{e^{y_{k}^{*}}}{\sum_{l=1}^{n}e^{y_{l}^{*}}}))$$

where n is the number of categories, $y_{i}^{*}$ is the final output, and $y_{i}$ is the ground truth. Combining Equations (2)–(4), the objective function of the proposed method is as follows:

(5) $$J=J_{1}+J_{2}+L_{c}$$

Algorithm 1 provides an overview of DHRL. The objective function of DHRL in Equation (5) can be optimized by using a stochastic gradient descent optimization algorithm.
**Algorithm 1.** Framework of the proposed DHRL method.**Input**: The training dataset $\Psi ={\left\{\left(x_{i}^{\alpha },x_{i}^{\beta }\right)\right\}}_{i=1}^{n}$ the label matrix Y, the dimensionality of common space d, the batch size $n_{b}$, the learning rate τ, and the maximal number of epochs ℵ. **Output:** Predict the label for the input image.
Randomly initialize the parameters of the two subnetworks ${ϒ}_{a}$ and ${ϒ}_{\beta }$, and the parameters of the linear classifier Q.For t=1,2,...,ℵ, doFor $l=1,2,\ldots ,[\frac{n}{n_{b}}]$, do   Randomly sample $n_{b}$ image pairs samples from Ψ to construct a mini-batch.   Compute the representations $u_{i}$ and $v_{i}$ for the samples in the mini-batch through forward propagation.   Calculate the result of the objective function in Equation (5).   Update the parameters of the subnetworks ${ϒ}_{a}$ and ${ϒ}_{\beta }$ and the linear classifier by minimizing the loss function.  End forEnd forCalculate the network output according to $Y^{\alpha *}=Q^{T}U$, $Y^{\beta *}=Q^{T}V$

## 4. Results

### 4.1. Datasets

NWPU-RESISC45 dataset [20]: Our proposed method was trained on the NWPU-RESISC45 dataset. This dataset was generated by the Northwestern Polytechnical University research team in 2017 and includes 31,500 remote sensing images and 45 scene classes. Each scene class consists of 700 images, where the dimensions of each image are 256 × 256. The spatial resolution of the majority of the images is 30~0.2 m/pixel, and some images of specific terrains may have a lower resolution, such as lakes, islands, and regular mountains. The dataset encompasses a diverse array of scene categories, with each category preserving substantial internal diversity while also displaying similarities to other categories.

AID dataset [34]: This dataset was created by Wuhan University in 2017. It has an image size of 600 × 600 and consists of 30 scene categories. Each category contains a maximum of 400 images and a minimum of 200 images, and the image resolution ranges from 0.5 to 8 m.

### 4.2. Implementation Details

In this work, our experimental verifications were carried out on a computer with a GTX TITAN GPU with 12G, and the algorithms were implemented with TensorFlow [35] and Keras [36]. The details of the parameter settings for the proposed DHRL were as follows. There were 30 epochs for the model, and the Adam [37] optimizer was applied with 10^−5^.

To assess the merits of our proposed algorithm in comparison with other state-of-the-art algorithms, it was imperative to ensure uniformity in data segmentation across all compared and benchmark methods. Therefore, using different training ratios for the different datasets allowed for a better analysis of the strengths and weaknesses of the method proposed in this study. For the NWPU-RESISC45 dataset, the training ratios were set to 10% and 20%, with the remaining 90% and 80% being used for testing. The training images were randomly rotated by 30° and flipped both horizontally and vertically. For the AID dataset, the training ratios were set to 20% and 50%, with the remaining 80% and 50% being used for testing. The training ratio represented the proportion of images in the dataset used for training. For example, 10% meant that 10% of the images were used for training. For a fair comparison, we used the same training ratios as those of the state-of-the-art methods for the NWPU-RESISC45 dataset and the AID dataset.

### 4.3. Comparison with Other State-of-the-Art Methods

#### 4.3.1. NWPU-RESISC45 Dataset

For the NWPU-RESISC45 dataset, the method proposed in this study was compared with existing methods (CNN-CapsNet [38], SCCov [31], ADFF, Siamese ResNet50 [39], FDPResnet [40], DDRL-AM [41], SF-CNN [18], and HABFNet [42]). The results are presented in Table 1. The experimental results showed that the test accuracy of our proposed method was 96.03 and 96.32 when the training ratio was 10% and 20%, respectively. CNN-CapsNet took full advantage of both CNN and CapsNet models, and its overall accuracy was 7% and 3.72% lower than that of the proposed DHRL method at the 10% and 20% training ratios. SCCov had accuracies of 89.30 and 92.10 at training ratios of 10% and 20%, which were 6.73% and 4.22% lower than those of DHRL. ADFF employed an attention mechanism, and its accuracy was 5.45% and 4.41% lower than that of DHRL. Siamese ResNet50 combined CNN identification and validation models, and it reduced the accuracy by 4.04% at a training ratio of 20%. FDPResnet is a fusion of the DCNN and the new and effective Extensive Learning System (BLS) for fast depth perception networks. It reduced the accuracy by 3.71% and 0.92% compared to DHRL. DDRL-AM [41] implemented depth-differentiated representation learning based on attention maps, and its accuracy was 3.86% lower than that of the method proposed in this study. HABFNet was a variety of methods based on the feature fusion framework of hierarchical attention and bilinear pooling. The proposed method improved upon its accuracy by 3.28% and 1.78%. Thus, the performance of DHRL on the NWPU-RESISC45 dataset was more effective than the performance of the current advanced methods, which confirmed the effectiveness of DHRL.

**Figure 4:**
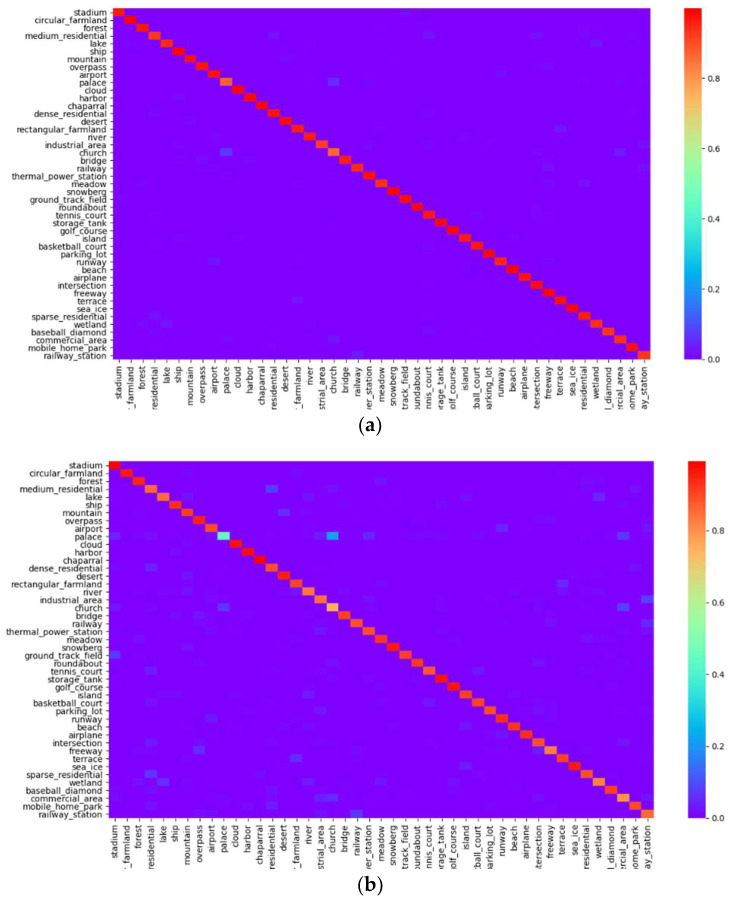
Confusion matrix of the proposed DHRL method on the NWPU-RESISC45 dataset. (**a**) Training ratio of 20%. (**b**) Training ratio of 10%.

To enhance the comprehension of the performance exhibited by DHRL, a confusion matrix was constructed to visually depict the accuracy of the classification. The result is shown in Figure 4. Each row in the matrix corresponds to the actual category, whereas each column pertains to the predicted category. Cells along the diagonal signify accurate predictions, while off-diagonal cells signify errors. The color within each cell denotes the cumulative count and percentage of prediction instances, with correct categorizations being arranged sequentially along the diagonal axis from left to right.

As shown in Figure 4, when the training ratio of the dataset was 20%, a classification accuracy of over 90% was achieved for most of the categories, which further indicated the effectiveness of DHRL. The classification accuracy was also obtained for categories with intra-category diversity, such as train stations and churches. For categories with inter-category similarity, such as churches and palaces, airports and lawns, and tennis and basketball courts, the classification accuracy was lower than that for other categories, but the error rate was low, indicating that DHRL was able to effectively solve the problem of intra-category diversity and inter-category similarity.

#### 4.3.2. AID Dataset

As shown in Table 2, the proposed DHRL method also yielded excellent results on the AID dataset. At 20% and 50% training ratios, the accuracy of DHRL was 93.08% and 96.54%, respectively. When the training ratio was 20%, DHRL outperformed most methods, improving the accuracy by 0.72%, 0.88%, and 2.83% compared to DDRL-AM [41], GBNet [46], and VGG_VD16+SAFF [47], and it had a similar accuracy to that of SCCov. ADFF represents an attention-based deep feature fusion framework comprising three key components: attentional mapping guided by gradient-weighted class activation mapping (GradCAM), multiplicative fusion of deep features, and the utilization of a center-based cross-entropy loss function. It was superior to our proposed DHRL method. However, at a 50% training ratio, DHRL outperformed all existing comparison methods, improving the accuracy by 0.44%, 1.09%, 1.79%, 4.74%, 1.06%, and 2.71% compared to SCCov [31], FACNN [27], ADFF, MCNN [42], GBNet [46], and VGG_VD16+SAFF [47]. When there was a small amount of training data, the multiple feature fusion strategy of ADFF was able to obtain more information for classification and achieve a slightly higher performance. When there was a large amount of training data, the confusion loss optimization strategy of the DHRL method was able to learn more discriminative representations for the samples that could be easily misclassified; therefore, the performance of DHRL was the best at a training ratio of 50%. Thus, the advantage of DHRL was more pronounced in the case of a larger amount of training data.

### 4.4. Ablation Study

To investigate the effectiveness of the proposed method, we designed experiments to evaluate the performance of the three losses ($J_{1}$, $J_{2}$, and $L_{c}$). The loss function of the DHRL method consisted of three components, which were used to characterize the discrimination loss in the common space, the discrimination loss in the label space, and the confusion loss of the classification. We developed three variations of the objective function of the proposed DHRL method: DHRL1 without $J_{1}$, DHRL2 without $J_{2}$, and DHRL3 without $L_{c}$. We evaluated the performance of these variations on the NWPU-RESISC45 dataset at training ratios of 10% and 20%. Table 3 shows the experimental classification results.

The result in Table 3 demonstrates that the performance of the full DHRL method was the best, which indicated that all three components of the loss function contributed to the scene image classification accuracy. We also observed that the reduction in the classification accuracy of DHRL2 was the largest because the second component $J_{2}$ optimized the discrimination loss directly in the label space. Based on the experimental results, it was proven that optimizing both the discrimination loss and confusion loss in the objective function was an effective learning method.

## 5. Conclusions

(1) In this study, we proposed a dual hierarchical representation learning approach for the problem of large dissimilarities in the same class and small dissimilarities between different classes in remote sensing image classification with the aim of exploring the dual hierarchical space, including a common space and a label space, to learn differentiated representations for remote sensing image classification by assessing the distinctions between different classes within the common space.
(2) Experimental evaluations conducted on challenging datasets demonstrated that the outcomes of the method proposed in this study surpassed those of existing state-of-the-art methods. This substantiated the efficacy and validity of the proposed approach.
(3) In our future work, we will extend the proposed method to multi-source remote sensing image classification. Fusing multi-source image features in the common space is a valuable strategy.

## Figures and Tables

**Table 1:** Classification accuracy of different methods on the NWPU-RESISC45 dataset.

Methods	Training Ratio (%)	
	10%	20%
CNN-CapsNet [38]	89.03	92.6
SCCov [31]	89.30	92.10
ADFF	90.58	91.91
Siamese ResNet50 [39]	—	92.28
FDPResnet [40]	92.32	95.40
DDRL-AM [41]	92.17	92.46
SF-CNN [18]	89.89	92.55
HABFNet [42]	92.75	94.54
RCOVBOVW [43]	90.25	93.27
HFFCNN [44]	87.01	90.14
MGSNet [45]	92.4	94.57
**DHRL (ours)**	**96.03**	**96.32**

**Table 2:** Classification accuracy of different methods on the AID dataset.

Methods	Training Ratio (%)	
	20%	50%
SCCov [31]	93.12	96.10
FACNN [27]	—	95.45
ADFF	**93.68**	94.75
DDRL-AM [41]	92.36	—
MCNN [42]	—	91.80
GBNet [46]	92.20	95.48
VGG_VD16+SAFF [47]	90.25	93.83
HFFCNN [44]	93.08	95.32
DHRL (ours)	93.08	**96.54**

**Table 3:** Classification accuracy of the DHRL method and its three variations on the NWPU-RESISC45 dataset.

Methods	Training Ratio (%)	
	10%	20%
DHRL1	93.22	94.53
DHRL2	82.19	85.67
DHRL3	91.35	92.82
**Full DHRL**	**96.03**	**96.32**

## Data Availability

Two datasets were used in this work: The NWPU-RESISC45 dataset is openly available at https://gcheng-nwpu.github.io/#Datasets accessed on 1 May 2022, and the AID dataset is openly available at https://captain-whu.github.io/AID/ accessed on 1 May 2022.
